# Use of Alternative Tobacco Products in Multiethnic Youth from Jujuy, Argentina

**DOI:** 10.1155/2010/795265

**Published:** 2010-03-16

**Authors:** Ethel Alderete, Celia Patricia Kaplan, Steven E. Gregorich, Eliseo J. Pérez-Stable

**Affiliations:** ^1^Instituto de Ciencia y Tecnología Regional, El Carmen 719, S.S. de Jujuy, 4600, Argentina; ^2^Consejo Nacional de Investigaciones Científicas y Técnicas (CONICET), Universidad Nacional de Jujuy, Argentina; ^3^Department of Medicine, University of California, San Francisco, 3333 California Street, Suite 335 San Francisco, CA 94118, USA; ^4^UCSF Helen Diller Family Comprehensive Cancer Center, USA

## Abstract

This study examines alternative tobacco use among Latin American youth. A self-administered survey in a random sample of 27 schools was administered in 2004 in Jujuy, Argentina (*N* = 3218). Prevalence of alternative tobacco product use was 24.1%; 15.3% of youth used hand-rolled cigarettes, 7.8% smoked cigars, 2.3% chewed tobacco leaf and 1.6% smoked pipe. Among youth who never smoked manufactured cigarettes, alternative product use was rare (2.9%), except for chewing tobacco (22%). In multivariate logistic regression boys were more likely than girls to smoke pipe (OR = 3.1; 95% CI 1.1–8.7); indigenous language was associated with smoking hand-rolled cigarettes (OR = 1.4; 95% CI-1.1–1.9) and pipe (OR = 2.2; 95% CI 1.5–3.4). Working in tobacco sales was a risk factor for chewing tobacco (OR = 2.9; 95% CI: 1.7–4.9) and smoking hand-rolled cigarettes (OR = 1.4; 95% CI 1.1–1.8). Having friends who smoked was associated with chewing tobacco (OR = 1.8; 95% CI 1.0–3.2) and with smoking cigars (OR = 2.1; 95% CI 1.5–2.9). Current drinking and thrill-seeking orientation were associated with cigars and pipe smoking. Findings highlight the importance of surveillance of alternative tobacco products use and availability among youth and for addressing identified risk factors.

## 1. Introduction

Alternative tobacco products may include hand-rolled cigarettes, cigars, processed or leaf tobacco for chewing, or regular or water pipes. These forms of tobacco consumption can deliver higher doses of tar, nicotine, and carbon monoxide than the standard manufactured cigarette and are also associated with adverse health effects [1, 2] including several types of cancers [3, 4], chronic obstructive pulmonary disease [5] coronary heart disease [6], gum recession, and nicotine addiction [7–10]. 

Few studies have documented the use of alternative tobacco products in Latin America where their use may be influenced by particular cultural and social characteristics. The tobacco plant originated in the Andean foothills of South America and the cultivation and the use of tobacco in different forms, by chewing, drinking, licking, snuffing, and smoking has been well documented and constitutes an ancestral tradition in Latin America [11]. On the other hand, contemporary use of alternative tobacco products may be influenced by the industry's promotion of products like cigars, hand rolled cigarettes, and smokeless tobacco as trendy choices. According to the Global Youth Tobacco Survey [12] the rate of current use of tobacco products other than cigarettes in the Americas (11,3%) is similar to the rest of the world [13]. 

We previously reported a detailed analysis of factors associated with current cigarette smoking in a representative sample of 3131 youth, 13 to 15 years of age, from Jujuy, Argentina [14] and found that Indigenous ethnicity doubled the odds of smoking in the previous 30 days (current smoking). Other risk factors that were associated with current smoking included having any friends who smoke, repeating a grade in school, depressive symptoms in previous year, drinking any alcohol in the previous week, and thrill seeking orientation. This study is the first to examine patterns and types of alternative tobacco use among Latin American youth.

## 2. Methods

### 2.1. Setting and Sampling Design

The study was conducted in the Province of Jujuy in northwest Argentina where about 44% of the population lives below the poverty level, the unemployment rates have been as high as 25% [15] and tobacco farming is one of the main economic activities in this region. Secondary schools were randomly sampled from within the three geographic areas of the province: the mountain region, the provincial capital, and the agricultural lowlands. Secondary schools include 8th through 12th grades and reflect the standard private and public school organization in Argentina. Based upon government data in 2004, we estimated the number of eighth grade students within each region to equal 1509, 7296, and 5379, respectively. The goal was to select a representative sample of schools containing approximately 1000 eighth grade students from within each geographic area (i.e., disproportionate stratification). The final sample included 27 schools, 3 of which were private [14]. We provided modest incentives to the schools in the form of supplies and training. There was no monetary compensation to youth or schools for collaboration.

### 2.2. Study Procedures

Spanish-language surveys were administered sequentially to schools between June and August of 2004, and within each sampled school we attempted to recruit every enrolled eighth grade student into the study. All surveys were conducted in Spanish. Surveys were self-administered in class with research staff and school coordinators present as proctors. In each school, one attempt was made to survey absent students on a subsequent date. The UCSF Committee on Human Research and an NIH-certified human subjects research board in Buenos Aires based at *Centro de Educación Médica e Investigaciones Clínicas *approved the protocol. Passive consent was requested from parents or caretakers and students signed an active consent form to allow follow-up contact for subsequent surveys.

### 2.3. Questionnaire Measures

The questionnaire included translations of items used in surveys of adolescents in the US [12] and items developed from our previous qualitative research. Items in English were translated and reviewed by three Argentinean investigators and two other Spanish-speaking research staff. Pilot testing of the instrument was conducted with students in rural and urban areas evaluating situational factors, content, characteristics of the respondents, and time of administration that averaged one hour. 

Smoking behavior questions included age at smoking initiation, number of cigarettes smoked in the lifetime, in the past 30 days, and in the past week, how many days in the past month and past week respondents smoked. Respondents were considered ever smokers if they tried at least a cigarette puff in their lifetime and never smokers had not tried even one puff. Current smokers were defined as having smoked at least one whole cigarette in their lifetime and at least one puff in the previous 30 days. Established smokers were defined as current smokers who had smoked at least 100 cigarettes in their lifetime. Use of tobacco products other than cigarettes was ascertained by asking students if they had ever smoked hand-rolled cigarettes, cigars, or pipes. We also asked whether respondents had used tobacco leafs for chewing. 

Exposure or predictor variables included demographic, family, and school characteristics. Respondents reported their sex, date of birth, and age, and if they were currently working or had ever worked in tobacco farming or sales. Ethnic self-identification was ascertained by providing a list of five ethnic categories to choose from: Indigenous, Mixed Indigenous and European (referred to as Mixed), European, Arab, and Other. Family characteristics included parent's formal education and employment status and family members speaking an indigenous language. We ascertained school location (rural, small town, urban), shift (morning, afternoon, evening), and type (public or private). 

Students reported on psychosocial risk factors, including the presence of smokers in the household, the number of friends who smoked, repeating a grade, and drinking at least one glass of alcohol in the previous week. Depressive symptoms were ascertained by asking a single item on whether the respondent in the past year felt sad and could not carry on his/her normal activities or obligations for at least 2 weeks [16]. The risk behavior orientation measure was adapted from one used with youth living in Florida and was measured with a five-point disagreement-agreement scale to three statements: “I do not mind getting in trouble as long as I have fun”, and “I like to do dangerous things”, “I like to do things that people say should not be done.” The measure had a range of 1 to 5 and we defined mean scores of 3 to 5 as high on thrill seeking and less than 3 as low on thrill seeking and thus adapted the measure to use as a binary version of the scale [17].

### 2.4. Data Analysis

The sampling design was incorporated into all models by specifying geographic areas as strata and schools as clusters as well as including weights to adjust for disproportionate stratification. In addition, a finite population correction was applied to adjust for the relatively large proportion of available schools sampled within each geographic area. Standard errors and confidence intervals were estimated via the Taylor expansion approximation [18]. 

Primary research questions included estimating total prevalence of alternative tobacco product use. First, we conducted descriptive analyses to profile the sample examining the distribution of demographic, family, and school characteristics and psychosocial risk factors by sex. Bivariate contingency tables examined the pairwise relationships among tobacco product use and social and demographic variables and psychosocial risk factors. Chi-square tests and *P* values were calculated. 

Multivariate logistic models regressed alternative tobacco product use (tobacco leafs, hand-rolled cigarettes, cigars, pipe) onto social and demographic variables (sex, age, ethnic identity, use of indigenous language, religion, currently working, ever working in tobacco farming, ever working in tobacco sales), family (parental education and employment status), school characteristics (location, type, and shift), cigarette smoking status (ever smoking and current smoking), and psychosocial risk factors (adult smokers at home, friends smoking, single parent household, repeating a grade, thrill seeking orientation, depressive symptoms, and current drinking). We estimated adjusted odds ratios and 95% confidence intervals.

## 3. Results

### 3.1. Participation Rate and Exclusions

The 27 participating schools included a total of 4276 registered eighth-grade students. Of those, 262 (6.1%) were absent on the days we attempted to recruit students into the study, and 324 (7.6%) declined participation, leaving 3690 (86.3%) participants who completed the questionnaire. Within each geographic stratum the participation rates were 81.5%, 84.3%, and 91.5% for the mountain, capital, and lowlands regions, respectively. The analysis was focused on under-age adolescents and therefore the sample was restricted to the 3526 students who were aged 12 to 17 years. Because categorization by ethnic identity was central to the analysis, the few respondents who self reported as Arabs (*n* = 28) or with other ethnicity (*n* = 12) and those with missing data for ethnicity (267) were excluded. Therefore the final sample included 3218 students.

### 3.2. Demographic Characteristics

The demographic characteristics of our sample were the following. Girls constituted about half of the sample (53%) and 82.5% of the respondents were 12–14 years of age. The majority of respondents were of indigenous or mixed indigenous/European ethnicity and only 7.5% identified as of European descent. Over 30% reported an indigenous language spoken in the family. One third of respondents (33.9%) were involved in the workforce at the time of the interview, 10.7% of girls and 14.9% of boys had ever worked in tobacco production, and 6.7% of girls versus 7.4% of boys had worked in tobacco sales. About 41% of parents had elementary school education or less and 22.6% were unemployed or recipients of social welfare. The majority of youth attended public schools (87.7%), and 34.5% attended schools in small towns or rural areas. At the time of the interview a similar proportion of girls and boys were ever smokers (49.8% versus 53.2%), current smokers (21.2% versus 24.1%), or established smokers (6.0% versus 7.5%). The majority 76.3% lived with an adult smoker, almost half (48.7%) had 5 or more friends who smoked, and 30.3% lived in single parent households. Furthermore, 43.6% reported having depressive symptoms, 25.4% repeated a grade, 17.4% showed a thrill seeking orientation, and 15.1% were current drinkers.

### 3.3. Alternative Tobacco Product Use

The prevalence of any alternative tobacco product consumption was 24.1% (Table 1). Hand-rolled cigarettes (15.3%) and cigars (7.8%) were the products used by most of respondents, followed by chewing of tobacco leaf (2.3%), and smoking a pipe (1.6%). Consumption of any tobacco product was higher among boys (27.1%) and youth of the older age group (15–17 years), among those who reported an indigenous language spoken in the family (30.8%), those who were currently working (34.5%) or who ever worked in tobacco production (35%) or sales (38.7%), and among those attending night school (39.0%). Living with an adult smoker (26.8%), having 5 or more friends who smoke (34.8), living with a single parent (26.8%), repeating a grade (34.0%), having a thrill seeking orientation (36.9%), reporting depressive symptoms (30.4%), or current drinking (47.8%) were also associated with more alternative tobacco use. Youth's ethnicity, religion, parent education and employment status, and school location were not related to alternative tobacco product use. 

The type of alternative tobacco product consumed defined groups of youth with different demographic characteristics. Girls and boys consumed all products in similar proportions except pipe (0.7% versus 2.8%). A higher percentage of older youth consumed hand-rolled cigarettes (23.6% versus 14.3%) and cigars (12.4% versus 7.7%) and a higher percentage of youth who reported an indigenous language spoken in their family smoked hand-rolled cigarettes (21% versus 13.4%) and pipe (2.5% versus 1.3%). Youth who ever worked in tobacco sales consumed more tobacco leaf (5.4% versus 2.0%), hand rolled cigarettes (28.4% versus 15%), and cigars (15.3% versus 7.9%). Attending a private versus a public school was associated with cigar smoking (10.7% versus 8.2%). The proportion of youth who used any of the four types of tobacco products was higher among those who had friends who smoke and among current drinkers. Currently working, repeating a grade and thrill seeking orientation was associated with smoking hand-rolled cigarettes, cigars, and pipe. Living with an adult smoker or in a single parent household and having depressive symptoms were associated with smoking hand-rolled cigarettes and cigars. 

Table 2 shows the use of alternative tobacco products by history of smoking manufactured cigarettes. Very few (2.9%) of the youth who had never tried manufactured cigarettes used other tobacco product, and almost half (47.4%) of youth who ever smoked cigarettes tried an alternative tobacco product. Almost all youth who reported use of hand-rolled cigarettes or cigars had smoked at some time, but a significant proportion (22%) of those using tobacco leaf reported never smoking a manufactured cigarette, followed by 8.2% of those smoking a pipe. Although the proportion defined as current smokers (past month) was over 50% for those using hand-rolled cigarettes, pipe, or cigars, only 35.2% of the 75 youth who chewed tobacco leaf were currently smoking.

### 3.4. Multivariate Analyses

In multivariate logistic regression models boys were more likely than girls to smoke pipe (OR = 3.1; 95% CI: 1.1–8.8) (Table 3). Indigenous language spoken in the family was significantly associated with smoking hand-rolled cigarettes (OR = 1.4; 95% CI: 1.1–1.9) and pipe (OR = 2.2; 95% CI = 1.5–3.4). Ever working in tobacco sales increased the likelihood of using tobacco leaf (OR = 2.9; 95% CI: 1.7–4.9) and of smoking hand rolled cigarettes (OR = 1.4; 95% CI: 1.1–1.8). Having 5 or more friends who smoked compared to having 1 to 4 was associated with tobacco leaf use (OR = 1.8; 95% CI: 1.0–3.2) and with smoking cigars (OR = 2.1; 95% CI: 1.5–2.9). Current drinking and having a thrill-seeking orientation were associated with cigars and pipe smoking.

## 4. Discussion

Our study is the first to report prevalence and risk factors by type of alternative tobacco product use among Latin American youth. Nearly one third of our sample had used tobacco products other than manufactured cigarettes, and almost half of those who were current smokers had used any alternative product. GYTS data (1999–2001) showed that the world mean of current use of other tobacco products was 8.8%, and the mean for the Americas was 11.3%. Boys were more likely than girls to use other tobacco products in the Americas, Europe, and South East Asia. No significant overall differences were reported by sex in other world regions. In addition, no sex differences were reported in 98 of the 147 sites [19]. A recent GYTS report analyzing changes between 1999 and 2008 shows that other tobacco use had increased in 34 world sites, and there was a significant increase between 2003 and 2007 for girls in Argentina [20]. However, GYTS does not provide data on lifetime use or on type of product used. In our sample, the overall use of other tobacco products was more likely to occur among boys. However, after controlling for social, demographic, and psychosocial factors, no significant differences were found between boys and girls for using tobacco leaf, or for smoking hand-rolled cigarettes or cigars. Similarly, although the overall proportion of younger youth who used other tobacco products was lower, age was not a significant risk factor after adjustment for other variables, except for smoking cigars. 

Tobacco leafs for chewing was the only smokeless product examined in this study. The use of tobacco leafs, easily available in this tobacco growing region, could constitute and appealing practice among a population of largely low-income individuals with precarious working conditions. Nevertheless, only 2.3% of our sample reported using this product. This may be due to the common use of coca leaf as a chewing product. This practice is legal in Jujuy; and has strong and ancient cultural roots. The implementation of smoke free policies for indoor public places is incipient or nonexistent in Jujuy, thus it is unlikely that tobacco leafs for chewing are used as a substitute for cigarettes, where smoking is not allowed. 

In previous reports, youth in this sample who self identified as being indigenous had higher rates of manufactured cigarette smoking, compared with youth of European descent. They were also more likely to smoke cigarettes after controlling for other factors [14]. In the present report, no significant association was found for the alternative tobacco products examined and self-reported indigenous ethnicity, but indigenous language spoken in the family was associated with the use hand-rolled cigarettes and pipe. This is an indication that indigenous youth living in less acculturated households where use of an indigenous language is maintained are at increased risk. Furthermore, the ceremonial use of pipes among Indigenous Peoples in the region in the past has been documented by archeological findings. However there are no published reports of contemporary ceremonial pipe use, only key informant reports that merit further exploration. Future studies should ascertain whether adolescents use pipes exclusively for recreational purposes or if there is any association to ritual practices. 

Hand-rolled cigarettes constitute a cheaper alternative to commercial cigarettes and were more frequently used by working youth, and especially those in tobacco sales. Use of rolled cigarettes and cigars is almost exclusively seen among youth who have tried manufactured cigarettes. Preference for cigar smoking may be a trait of youth of higher social class and of European descent, since it was associated with attendance to a private school. This finding also merits further elucidation. Other factors associated with current smoking of manufactured cigarettes were also associated with cigar smoking such as having friends who smoke, alcohol drinking, a thrill seeking orientation, and having depressive symptoms [14]. 

Results indicate that the consumption of alternative tobacco products in this region may not only be due to popular trends [21] but also that socioeconomic and cultural factors play a significant role. Moreover, social and demographic characteristics define groups of at-risk youth, in particular girls, younger youth, and working and less acculturated Indigenous youth. Different types of tobacco products appear to be readily available. Additionally, the potential role of alternative tobacco use in facilitating the transition from experimenting to current smoking is a salient cause for concern that deserves further study. Our results show the importance of addressing socioeconomic and cultural aspects of behavior in tobacco control prevention in the region, the need to restrict accessibility of all types of tobacco products by youth, and to monitor the promotion of these products by the tobacco industry. These findings also highlight the importance of public health surveillance of alternative tobacco products use among youth and the significance of expanding the reach of studies that address the ethnic diversity and vast socioeconomic gaps in Latin America.

## Figures and Tables

**Table 1:** Lifetime alternative tobacco product use by demographic, social, and psychosocial risk factors. Youth 12 to 17 years of age, Jujuy, Argentina, 2004.

	*N* by Category	Tobacco Leaf	Hand-rolled Cigarettes	Cigars	Pipe	Any Alternative Tobacco Product
Total *N*	3218	*N* = 75	*N* = 519	*N* = 268	*N* = 56	*N* = 813
		(2.3%)	(15.3%)	(7.8%)	(1.6%)	(24.1%)
		% (s.e)	% (s.e)	% (s.e)	% (s.e)	% (s.e)
Youth Demographic Factors						

*Sex*						
Girls	1695	2.0 (0.3)	15.2 (1.6)	7.5 (0.8)	0.7 (0.3)*	23.0 (1.8)*
Boys	1523	2.4 (0.4)	16.7 (1.3)	9.7 (1.0)	2.8 (0.5)	27.1 (1.7)
*Age*						
12–14	2655	2.2 (0.3)	14.3 (1.2)*	7.7 (0.8)*	1.5 (0.3)	22.5 (1.4)*
15–17	563	2.3 (0.7)	23.6 (1.9)	12.4 (1.8)	2.6 (0.5)	36.2 (1.9)
*Ethnicity*						
Indigenous	2259	2.2 (0.3)	15.7 (1.6)	7.6 (0.8)	1.9 (0.3)	23.8 (1.8)
Mixed	714	1.9 (0.6)	18.6 (2.1)	9.9 (1.6)	1.1 (0.5)	28.5 (2.1)
European	241	3.3 (1.5)	10.4 (2.0)	12.7 (2.5)	0.6 (0.4)	24.6 (4.2)
*Indigenous Language Spoken in the Family*						
No	2195	2.0 (0.3)	13.4 (1.4)*	8.2 (0.8)	1.3 (0.2)*	22.2 (1.7)*
Yes	1023	2.6 (0.4)	21.0 (1.5)	9.5 (1.4)	2.5 (0.4)	30.8 (1.4)
*Working*						
No	2127	2.0 (0.3)	12.0 (1.1)*	7.2 (0.9)*	1.1 (0.3)*	19.8 (1.3)*
Yes	1023	2.8 (0.5)	23.1 (1.3)	11.2 (1.1)	2.8 (0.5)	34.5 (1.8)
*Ever Worked in Tobacco Farming*						
No	2809	2.0 (0.3)	14.9 (1.2)	8.1 (0.8)	1.6 (0.3)	23.4 (1.5)*
Yes	409	3.9 (1.1)	23.2 (1.6)	11.1 (1.8)	2.5 (0.6)	35.0 (2.0)
*Ever Worked in Tobacco Sales *						
No	2993	2.0 (0.2)*	15.0 (1.3)*	7.9 (0.7)*	1.6 (0.2)	23.9 (1.5)*
Yes	225	5.4 (1.3)	28.4 (2.2)	15.3 (3.1)	2.7 (1.0)	38.7 (3.3)

Parents Demographic Factors						

*Education*						
Elementary or less	1319	2.3 (0.5)	18.4 (1.4)	8.1 (0.9)	1.9 (0.3)	27.0 (1.9)
High-school	1091	2.0 (0.4)	14.7 (1.7)	6.9 (1.0)	1.3 (0.4)	22.4 (1.9)
Technical or College	808	2.1 (0.7)	14.1 (1.9)	11.3 (1.8)	1.7 (0.5)	25.0 (2.7)
*Employment Status*						
Unemployed	167	1.4 (0.9)	16.2 (2.4)	5.9 (2.1)	1.4 (0.8)	23.5 (2.7)
Social welfare	560	2.7 (0.8)	17.6 (1. 7)	8.0 (1.6)	0.9 (0.3)	26.2 (2.7)
Employed or retired	2488	2.1 (0.3)	15.6 (1.4)	8.7 (0.9)	1.9 (0.3)	24.6 (1.5)
School Characteristics						

*Type*						
Private	396	1.8 (0.8)	13.2 (0.9)	10.7 (0.6)*	1.4 (1.0)	24.3 (0.9)
Public	2822	2.3 (0.2)	16.3 (1.4)	8.2 (0.8)	1.7 (0.2)	25.0 (1.7)
*School Shift*						
Day	1751	2.5 (0.3)	14.7 (1.3)*	9.0 (1.0)	1.6 (0.4)	24.2 (1.7)*
Afternoon	1229	2.0 (0.6)	15.4 (1.7)	7.2 (1.2)	1.7 (0.3)	23.1 (2.5)
Night	238	1.5 (0.4)	27.3 (1.8)	11.7 (0.8)	2.0 (0.7)	39.0 (2.6)

Youth Psychosocial Risk Factors						

*Adult Smoker at Home*	2455	2.4 (0.3)	17.0 (1.5)*	9.4 (0.8)*	1.8 (0.3)	26.8 (1.8)*
*Friends Smoking*						
0	808	1.3 (0.4)*	6.7 (1.1)*	3.4 (0.9)*	0.4 (0.2)*	11.3 (1.5)*
1–4	846	1.5 (0.3)	15.3 (1.3)	5.3 (0.9)	1.3 (0.3)	21.9 (1.3)
5+	1564	3.2 (0.3)	21.9 (1.7)	13.4 (1.0)	2.5 (0.3)	34.8 (1.8)
*Single Parent Household*	975	2.4 (0.3)	17.0 (1.5)*	9.4 (0.8)*	1.7 (0.3)	26.8 (1.8)*
*Repeat Grade*	817	2.3 (0.5)	22.6 (1.5)*	10.7 (1.0)*	2.7 (0.3)*	34.0 (2.0)*
*Thrill Seeking Orientation*	560	3.3 (0.8)	23.8 (1.9)*	15.4 (1.8)*	4.9 (0.9)*	36.9 (2.2)*
*Depressive Symptoms*	1403	2.6 (0.5)	19.8 (1.3)*	10.8 (1.3)*	2.2 (0.4)	30.4 (1.5)*
*Current Drinker*	486	4.1 (0.9)*	30.8 (2.2)*	18.9 (1.9)*	5.0 (1.0)*	47.8 (2.6)*

*Significant at *P* < .05.

**Table 2:** Alternative tobacco product use by manufactured cigarette smoking status, Youth 12 to 17 years of age, Jujuy Argentina, 2004.

Alternative Tobacco Product Used	Cigarette Smoking Status			
	*N*	Never Smoker	Ever Smoker, Not Current	Current Smoker
Tobacco Leaf	75	22.0%	42.8%	35.2%
Hand-rolled Cigarettes	519	1.1%	46.7%	52.3%
Cigar	268	0.9%	44.2%	55.0%
Pipe	56	8.2%	30.9%	61.0%
Any Alternative Tobacco Product	813	2.9%	47.4%	49.7%

**Table 3:** Predictors of lifetime alternative tobacco product use. Youth 12 to 17 years of age, Jujuy, Argentina, 2004^#^.

	Tobacco Leaf	Hand-rolled Cigarettes	Cigars	Pipe
	OR (95% CI)	OR (95% CI)	OR (95% CI)	OR (95% CI)
Youth Demographic Factors				

*Sex*				
Boys versus Girls	1.3 (0.9–2.0)	0.9 (0.7–1.1)	1.0 (0.8–1.3)	3.1 (1.1–8.8)*
*Indigenous Language Spoken in the Family*				
Yes versus No	1.1 (0.7–1.8)	1.4 (1.1–1.9)*	1.2 (0.8–1.7)	2.2 (1.5–3.4)*
*Ever Worked in Tobacco Sale*				
Yes versus No	2.9 (1.7–4.9)*	1.4 (1.1–1.8)*	1.0 (0.6–1.8)	0.7 (0.3–1.6)
*Employment Status*				
Govt. assistance versus Employed/retired	1.4 (0.7–2.9)	1.0 (0.8–1.3)	0.9 (0.7–1.3)	0.5 (0.2–1.0)
Unemployed versus Employed/retired	0.9 (0.2–3.6)	1.0 (0.6–1.8)	0.8 (0.3–1.9)	0.9 (0.3–2.7)

School Characteristics				

*Type*				
Public versus Private	1.6 (0.6–4.3)	0.9 (0.6–1.3)	0.8 (0.5–1.6)	1.0 (0.2–4.6)
*Location*				
Small town versus Urban	0.7 (0.5–1.0)	1.3 (1.0–1.5)	0.7 (0.5–1.0)	0.7 (0.4–1.5)
Rural versus Urban	0.6 (0.3–1.1)	1.0 (0.6–1.5)	1.1 (0.6–2.0)	0.3 (0.1–1.0)*
*Shift*				
Day versus Afternoon	1.6 (0.7–3.3)	1.26 (1.0–1.6)	1.3 (0.9–1.9)	1.2 (0.5–2.6)
Day versus Night	4.3 (1.6–12.0)	1.2 (0.9–1.7)	1.3 (0.7–2.3)	1.5 (0.4–5.6)
Afternoon versus Night	2.8 (0.7*℃*11.7)	1.0 (0.7–1.3)	1.0 (0.6–1.7)	1.3 (0.5–3.8)

Youth Psychosocial Risk Factors				

*Household*				
2 Parents versus 1	1.3 (0.7–2.3)	0.8 (0.7–0.9)	0.9 (0.6–1.2)	1.4 (0.7–2.9)
*Adult Smoker at Home *	1.1 (0.6–1.9)	1.1 (0.9–1.4)	1.1 (0.7–1.6)	0.8 (0.4–1.6)
*Friends Who Smoke*				
1–4 versus 0	0.7 (0.3–1.5)	1.2 (0.8–1.8)	0.7 (0.5–1.1)	1.9 (0.7–4.6)
5+ versus 0	1.3 (0.7–2.5)	1.2 (0.8–1.8)	1.5 (0.9–2.5)	2.1 (0.8–5.9)
5+ versus 1–4	1.8 (1.0–3.2)*	1.0 (0.9–1.2)	2.1 (1.5–2.9)*	1.2 (0.8–1.7)
*Repeated a Grade*	0.9 (0.5–1.6)	1.1 (1.0–1.3)	1.1 (0.8–1.5)	1.2 (0.8–1.9)
*Thrill Seeking Orientation*	1.1 (0.6–2.0)	1.3 (1.1–1.6)	1.5 (1.1–2.0)*	3.7 (1.8–7.4)*
*Depressive Symptoms *	1.0 (0.6–1.8)	1.1 (0.9–1.5)	1.2 (0.9–1.7)	1.3 (0.7–2.3)
*Current Drinker *	1.4 (0.6–3.5)	1.2 (0.9–1.6)	1.5 (1.1–2.1)*	2.8 (1.3–6.2)*

^#^Controlling for ever smoking, youth's age, ethnicity, currently working, ever working in tobacco production, parent occupation and employment status, and type of school.
